# Presynaptic spike-driven plasticity based on eligibility trace for on-chip learning system

**DOI:** 10.3389/fnins.2023.1107089

**Published:** 2023-02-23

**Authors:** Tian Gao, Bin Deng, Jiang Wang, Guosheng Yi

**Affiliations:** School of Electrical and Information Engineering, Tianjin University, Tianjin, China

**Keywords:** spiking neural network, adaptive LIF model, eligibility trace, presynaptic spike-driven, on-chip learning system

## Abstract

**Introduction:**

Recurrent spiking neural network (RSNN) performs excellently in spatio-temporal learning with backpropagation through time (BPTT) algorithm. But the requirement of computation and memory in BPTT makes it hard to realize an on-chip learning system based on RSNN. In this paper, we aim to realize a high-efficient RSNN learning system on field programmable gate array (FPGA).

**Methods:**

A presynaptic spike-driven plasticity architecture based on eligibility trace is implemented to reduce the resource consumption. The RSNN with leaky integrate-and-fire (LIF) and adaptive LIF (ALIF) models is implemented on FPGA based on presynaptic spike-driven architecture. In this architecture, the eligibility trace gated by a learning signal is used to optimize synaptic weights without unfolding the network through time. When a presynaptic spike occurs, the eligibility trace is calculated based on its latest timestamp and drives synapses to update their weights. Only the latest timestamps of presynaptic spikes are required to be stored in buffers to calculate eligibility traces.

**Results:**

We show the implementation of this architecture on FPGA and test it with two experiments. With the presynaptic spike-driven architecture, the resource consumptions, including look-up tables (LUTs) and registers, and dynamic power consumption of synaptic modules in the on-chip learning system are greatly reduced. The experiment results and compilation results show that the buffer size of the on-chip learning system is reduced and the RSNNs implemented on FPGA exhibit high efficiency in resources and energy while accurately solving tasks.

**Discussion:**

This study provides a solution to the problem of data congestion in the buffer of large-scale learning systems.

## Introduction

Supervised learning is a training method for neural networks widely used in the fields of pattern recognition (Schwenker and Trentin, 2014), image processing (Aljuaid and Anwar, 2022), and semantic segmentation (Zhou et al., 2022), which is generally realized on the graphics processing unit (GPU) and the central processing unit (CPU). Due to the frequent data transmission between memories and process units, the GPU and the CPU are difficult to solve problems, such as high energy consumption and high demand for hardware specifications. A series of hardware systems have been proposed to train neural networks more efficiently, which presents as low-power dissipation and less hardware resource utilization (Dundar et al., 2017; Kriegeskorte and Mok, 2017; Davies et al., 2018). In these neuromorphic systems, spiking neural networks (SNNs) are considered to be more suitable for digital circuits (Painkras et al., 2013; Merolla et al., 2014; Lechner et al., 2020). It is necessary to train the neural networks on neuromorphic hardware systems in a quick and energy-saving way for the applications in the terminal or edge equipment (Chu et al., 2015; Kornijcuk and Jeong, 2019; Shama et al., 2020). As an important type of SNNs, recurrent spiking neural networks (RSNNs) are considered difficult to be trained on chips because of the large number of parameters and complex dynamics. While training the RSNN, the network is usually unfolded through time, which makes a challenge for digital circuits. A typical example is the backpropagation through time (BPTT) algorithm, which is thought as a common learning algorithm used to train RSNNs (Werbos, 1990; Manneschi and Vasilaki, 2020). Although it has been proved to perform excellently in the fields including speech recognition (Ahmad et al., 2004; Tang and Glass, 2018) and phoneme recognition (Hermans et al., 2015), the full-time storage for variables and backpropagation through a long period of time are so luxury for on-chip memories. The complex gradients of RSNNs and the huge requirement for memories make it difficult to realize a learning system for RSNNs.

To solve this problem, a series of algorithms and architectures based on surrogate-gradients are proposed to train RSNNs on circuits in a hardware-friendly way. Zhang and Li (2019) optimize the computation of gradients without unfolding the network through time and performing backpropagation time point by time point, which relies on the architecture driven by time. Compared with the backpropagation on spike-train level, the local synaptic plasticity is expected to apply on RSNNs, which consumes fewer computations and memories (Larsen and Sjöström, 2015; Kaiser et al., 2020). Bellec et al. (2019, 2020) propose the eligibility backpropagation (e-prop) algorithm to replace unfolding the RSNN through time by the surrogate-gradient based on eligibility traces, which is known as the fading memory of events (Liu et al., 2020; Kalhor et al., 2021). Benefit from the local learning in synapses, the e-prop algorithm is considered suitable for mapping to the circuits like field programmable gate array (FPGA). However, the buffer size is related to the length of trace, which is used to cache the fading memory of events in the time window (Fieres et al., 2008; Millner et al., 2010; Benjamin et al., 2014). It results in that the requirement of buffer size is not only linearly related to the size of neuron array, but also exponentially related to dynamic network activity. This problem is widespread in time-driven architectures (Moore et al., 2012; Pani et al., 2017). Park and Jung (2020) propose a presynaptic spike-driven spike timing-dependent plasticity (STDP) learning rule in the address domain. This method provides a way to trace spike trains based on timestamps and synaptic update rates in a STDP time window, instead of storing the complex relationship between presynaptic and postsynaptic spikes. Inspired by this, the presynaptic spike is possible used to trigger calculations of eligibility traces based on timestamps. In this way, the buffer size can be reduced and the learning system works with less on-chip memory consumption.

In this study, we aim to realize a high-efficient RSNN learning system on FPGA. We implement the RSNN based on the presynaptic spike-driven architecture to optimize synaptic modules. When a presynaptic spike occurs, it activates the synaptic module to search the eligibility trace value based on the latest timestamp. Based on this architecture, the buffer size of the on-chip learning system is reduced. We show this high-efficient implementation and test it on two experiments. The classification and synthesis results confirm that the RSNN reaches a satisfactory accuracy and efficiency in resources and energy consumption. This architecture provides a solution for the large amounts of data transferred and stored in the buffers of large-scale neuromorphic systems.

## Materials and methods

The RSNNs tend to have inferior short-term memory capabilities, which leads to weaker learning abilities in sequential tasks. Bellec et al. (2018) use the RSNN with the leaky integrate-and-fire (LIF) and adaptive leaky integrate-and-fire (ALIF) models to enhance the short-term memory, which improves the performance of RSNNs in sequential tests. In this study, we implement the RSNN proposed by Bellec et al. (2018) on FPGA as an on-chip learning system. Considering that the synaptic modules of the ALIF models implemented on FPGA require more logic elements than LIF models, we find a balance between the accuracy and resource consumptions by changing the ratio of the numbers of two models. Further, the RSNN includes the inhibitory and excitatory neurons, which limits the synaptic weights to (−1, 0] and [0, 1) to match the input range of the multipliers in synaptic modules. In the implementation of the RSNNs on FPGA, the presynaptic spike-driven architecture is applied to the synaptic modules, which contributes to the reduction of buffer size. With this architecture, a high-efficient on-chip learning system based on the RSNN is realized on FPGA.

### ALIF model with SFA mechanism

In the ALIF model, spike-frequency adaptation (SFA) based on the dynamic threshold is applied to the LIF model (Benda and Herz, 2003; Wang et al., 2003; Bellec et al., 2018, 2020; Salaj et al., 2021). The membrane potential of LIF models is calculated as:


(1) $$v_{j}^{t}=\alpha \,v_{j}^{t-\Delta \,t}+\sum_{i}W_{j\,i}^{I\,n}\,x_{i}^{t-d}+\sum_{i}W_{j\,i}^{R\,e\,c}\,z_{i}^{t-d}-z_{j}^{t-\Delta \,t}\,v_{t\,h\,r}$$

(2) $$\alpha =e^{-\Delta \,t/\tau_{v}}$$

where *v_j_^t^* is membrane potential of the *j*th neuron in hidden layer at time *t*, α is the attenuation constant of membrane potential, *W_ji_^In^* is input synaptic weight from the *i*th neuron in input layer to the *j*th neuron in hidden layer, *x_i_^t–d^* is input spike from the *i*th neuron in input layer at time *t*–*d*, *W_ji_^Rec^* is recurrent synaptic weight from the *i*th neuron in hidden layer to the *j*th neuron in hidden layer, *z_i_^t–d^* is the spike output by the *i*th neuron in hidden layer at time *t*–*d*, *z_j_^t–Δt^* is the spike output by the *j*th neuron in hidden layer at time *t*–Δ*t*, *d* is the transmission delay, *v_thr_* is the threshold voltage, Δ*t* is the timestep, and *τ_v_* is the time constant of membrane potential. If the membrane potential reaches the threshold, the LIF model generates a spike and then enters a refractory period.

The dynamics of membrane potential in ALIF models are similar to LIF models. The ALIF model has another state variable besides the membrane potential. The basic threshold voltage of ALIF models is equal to the threshold voltage of LIF models. With continuous activated by input currents, the threshold voltage of ALIF models increases rapidly. If the membrane potential of ALIF model is below the threshold for a long time, the threshold voltage gradually decreases to the basic value. The dynamic threshold voltage is described as:


(3) $$B_{j}^{t}=b^{b\,a\,s\,e}+\beta \,b_{j}^{t}$$

(4) $$b_{j}^{t}=\rho \,b_{j}^{t-\Delta \,t}+\left(1-\rho \right)\,z_{j}^{t-\Delta \,t}$$

(5) $$\rho =e^{-\Delta \,t/\tau_{a}}$$

(6) $$z_{j}^{t}=H\,\left(v_{j}^{t}\right)$$

where *B* is the threshold voltage, *b^base^* is the basic value, β is the scaling factor, ρ is the attenuation constant of threshold voltage, *τ_*a*_* is the time constant of dynamic threshold voltage, and *H*(*x*) is the Heaviside function.

### Eligibility trace in synapses

The eligibility trace is a temporary trace of events generated by neurons. It combines the gradient at present and in the past to update synaptic weights and reduce the gradient of RSNNs (Sutton and Barto, 2014). Compared with BPTT algorithm, which has performed excellently in RNNs, the eligibility trace allows neurons to store local gradients instead of backpropagating through time and area. Because spikes are differentiable impulse signals, the pseudo-derivative function is used to described the derivative of spikes. The pseudo-derivative function is calculated as (Bellec et al., 2018):


(7) $$\frac{d\,z}{d\,v}=\gamma \,\max\left\{0,1-\left|\frac{v-b^{b\,a\,s\,e}}{b^{b\,a\,s\,e}}\right|\right\}$$

where Υ is the pseudo-derivative of amplitude. When the neuron is in refractory period, the pseudo-derivative is set to 0. For ALIF model, the derivative of Heaviside function is defined as:


(8) $$\frac{d\,z}{d\,v}=\gamma \,\max\left\{0,1-\left|\frac{v-B}{b^{b\,a\,s\,e}}\right|\right\}$$

The eligibility trace is based on the presynaptic neuron. An internal variable vector *h^t^*∈ *R* is assumed as the states of dynamics in models. The eligibility trace is defined as following:


(9) $$e_{j\,i}^{t}=\frac{d\,z_{j}^{t}}{d\,h_{j}^{t}}\,\varepsilon_{j\,i}^{t}$$

(10) $$\varepsilon_{j\,i}^{t}=\frac{\partial h_{j}^{t}}{\partial h_{j}^{t-1}}\,\varepsilon_{j\,i}^{t-1}+\frac{\partial h_{j}^{t}}{\partial W_{j\,i}}$$

In the LIF model, *h_*j*_*^t^** is a one-dimension vector, which includes the membrane potential *v_*j*_*^t^**. The eligibility trace in LIF models is calculated as:


(11) $$e_{j\,i}^{t}=\frac{d\,z_{j}^{t}}{d\,v_{j}^{t}}\,{\bar{z}}_{i}^{t-d}$$

(12) $${\bar{z}}_{i}^{t}=\sum_{t-d\leq t^{\prime }\leq t}\alpha^{t-t^{\prime }}\,z_{i}^{t^{\prime }}$$

where *e_ji_^t^* is the eligibility trace of synapse from the *i*th neuron to the *j*th neuron, and α*^t–t′^* is the attenuation constant of membrane potential that decays over time. For input synaptic weights *W^In^*, the output of neurons *z* is replaced by inputs *x*.

In the ALIF model, *h_j_^t^* consists two dimensions, i.e., the membrane potential *v_j_^t^* and the dynamic threshold voltage *B^t^*. The derivative of *h_j_^t^* is a 2 × 2 matrix described as:


(13) $$\frac{d\,h_{j}^{t}}{d\,h_{j}^{t-1}}=\left[\begin{array}{cc} \frac{\partial v_{j}^{t}}{\partial v_{j}^{t-1}} & \frac{\partial v_{j}^{t}}{\partial b_{j}^{t-1}}\\ \frac{\partial b_{j}^{t}}{\partial v_{j}^{t-1}} & \frac{\partial b_{j}^{t}}{\partial b_{j}^{t-1}} \end{array}\right]=\left[\begin{array}{cc} \alpha & 0\\ \beta \,\frac{d\,z_{j}^{t-1}}{d\,v_{j}^{t-1}} & \rho -\beta \,\frac{d\,z_{j}^{t-1}}{d\,v_{j}^{t-1}} \end{array}\right]$$

The eligibility trace in ALIF models is calculated as:


(14) $$e_{j\,i}^{t}=\frac{d\,z_{j}^{t}}{d\,v_{j}^{t}}\,\left({\bar{z}}_{i}^{t-d}-\beta \,\varepsilon_{j\,i}^{t}\right)$$

(15) $$\varepsilon_{j\,i}^{t}=\left(\rho -\beta \,\frac{d\,z_{j}^{t-1}}{d\,v_{j}^{t-1}}\right)\,\varepsilon_{j\,i}^{t-1}+\frac{d\,z_{j}^{t-1}}{d\,v_{j}^{t-1}}\,{\bar{z}}_{i}^{t-d-1}$$

### Synaptic plasticity

In this study, the RSNN is composed of ALIF and LIF models. Connections between neurons are sparsely with a constant connection probability 60%. The filtered and weighted outputs of the RSNN are used as predictions, which are described as:


(16) $$y_{j}^{t}=\left(1-\lambda \right)\,\sum_{t-d\leq t^{\prime }\leq t}\sum_{i}\lambda^{t-t^{\prime }}\,W_{j\,i}^{O\,u\,t}\,z_{i}^{t^{\prime }}+b_{j}^{O\,u\,t}$$

(17) $$\lambda =e^{-\Delta \,t/\tau_{o\,u\,t}}$$

where *y* is the output of RSNN, λ is the attenuation constant of outputs, *λ^t–t′^* is the attenuation constant that decays over time, *W_ji_^Out^* is output synaptic weight from the *i*th neuron in hidden layer to the *j*th neuron in output layer, *b_j_^Out^* is output bias of the *j*th output node, and *τ_*out*_* is the time constant of outputs. The SoftMax function is used to activate the predictions. The output node with the maximum value is the predicted label.

During the training period, the gradient is divided into two parts: the eligibility trace and the learning signal (Bellec et al., 2020). As described before, the eligibility trace updates synaptic weights towards historical gradients. A learning signal guides the RSNN to minimize errors between predicted targets and real targets. The learning signal contains errors based on the loss function, which is used to evaluate the performance of RSNNs defined as:


(18) $$L_{i}^{t}=\sum_{j}W_{i\,j}^{B\,a\,c\,k}\,\left(Y_{j}^{t}-Y_{j}^{*t}\right)$$

where *L_*i*_*^t^** is the learning signal of the *i*th neuron in the hidden layer at time *t*, *W_*ji*_*^Back^** is feedback synaptic weights from the *j*th neuron in output layer to the *i*th neuron in hidden layer, *Y_*j*_*^t^** is predicted target of the *j*th output node at time *t*, and Y_j_^*t^ is real target of the *j*th output node at time *t*. Gradients of input and recurrent synaptic weights are defined as:


(19) $$\frac{d\,E}{d\,W_{j\,i}}=\sum_{t_{1}}\frac{d\,E}{d\,h_{j}^{t_{1}}}\,\frac{\partial h_{j}^{t_{1}}}{\partial W_{j\,i}}$$

(20) $$\frac{\partial h_{j}^{t}}{\partial W_{j\,i}}:=\frac{\partial z_{j}^{t}}{\partial h_{j}^{t}}\,\sum_{t_{1}\leq t}\frac{\partial h_{j}^{t}}{\partial h_{j}^{t-1}}\,\frac{\partial h_{j}^{t-1}}{\partial h_{j}^{t-2}}\,\ldots \,\frac{\partial h_{j}^{t_{1}}}{\partial W_{j\,i}}=\frac{\partial z_{j}^{t}}{\partial h_{j}^{t}}\,\varepsilon_{j\,i}^{t}=e_{j\,i}^{t}$$

(21) $$\begin{array}{ccc} \frac{d\,E}{d\,z_{i}^{t}} & = & \sum_{j}W_{i\,j}^{B\,a\,c\,k}\,\left(Y_{j}^{t}-Y_{j}^{*t}\right) \end{array}$$

The eligibility trace is restricted in a short time window and recurrent synaptic weights are updated as:


(22) $$W_{j\,i}^{R\,e\,c}=W_{j\,i}^{R\,e\,c}-\eta \,\sum_{t}L_{j}^{t}\,{\bar{e}}_{j\,i}^{t}$$

(23) $${\bar{e}}_{j\,i}^{t}=\sum_{t-d\leq t^{\prime }\leq t}\lambda^{t-t^{\prime }}\,e_{j\,i}^{t^{\prime }}$$

where η is the learning rate. The input synaptic weight *W_*ji*_*^In^** is updated same as *W_*ji*_*^Rec^**. The output weight *W_*ji*_*^Out^** is updated by the gradient descent algorithm. The cross entropy is used as the loss function. Output synaptic weights are updated as:


(24) $$W_{j\,i}^{O\,u\,t}=W_{j\,i}^{O\,u\,t}-\eta \,\sum_{t-d\leq t^{\prime }\leq t}\lambda^{t-t^{\prime }}\,z_{i}^{t^{\prime }}\,\left(Y_{j}^{t^{\prime }}-Y_{j}^{*t^{\prime }}\right)$$

**FIGURE 1:**
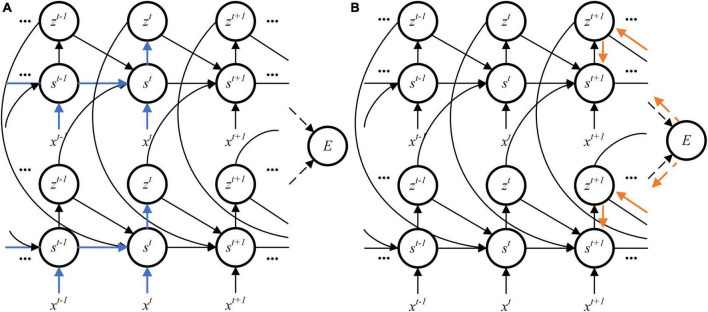
Data flow in gradient computation. **(A)** Eligibility trace. **(B)** Learning signal.

All parameters mentioned in this study are shown in Table 1 (Bellec et al., 2018). Different from BPTT algorithm, the synaptic plasticity used in this study only needs errors at present time. In contrast, synaptic weights are generally optimized at the end of training in BPTT algorithm. State variables during the entire training period are stored for gradients calculation. The requirement of on-chip memory is very luxury for FPGA. Figure 1A shows the data flow of the eligibility trace. It does not need to wait and store latent variables in the RSNN until the end of training. At each timestep, the eligibility trace is calculated and applied to gradients. Figure 1B shows the data flow of learning signals. The learning signal is corresponding to errors between predicted targets and real targets. At the end of training, synaptic weights are updated with the combination of eligibility trace and learning signal. With the eligibility trace gated by learning signal, the RSNN learns in a hardware-friendly way.

**TABLE 1:** Parameter values used in the RSNN.

Symbol	Value	Symbol	Value	Symbol	Value	Symbol	Value	Symbol	Value
Δ*t*	1	*b^base^*	0.01	*d*	5	β	1.8	*Ref*	5
η	0.01	*τ_*m*_*	20	Υ	0.3	*τ_*a*_*	500	*τ_*out*_*	20

### Architecture overview

**FIGURE 2:**
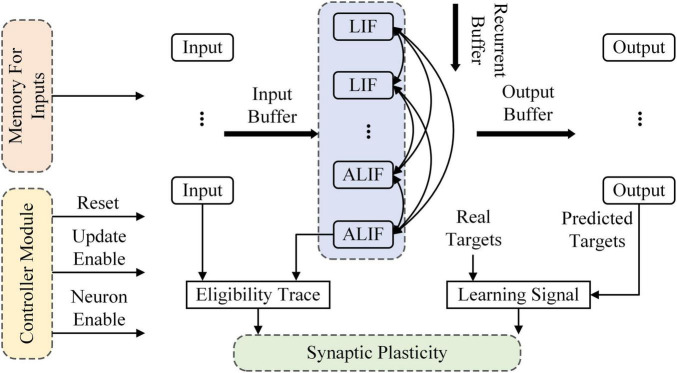
Overview of the RSNN architecture implemented on FPGA.

The RSNN used in this study is implemented on Altera Stratix V Advanced Systems Development Kit with Stratix V GX FPGA as an on-chip learning system. Figure 2 overviews the architecture of the RSNN, which is composed of a controller, memories for inputs, computing units and the synaptic plasticity block. The controller contains a counter used as system clock. At the beginning of training, the reset port is set to 1 and transmitted to all modules in the system. Then the enable port is set to 1 and the reset port is set to 0. The RSNN begins to receive inputs from memories and outputs predicted targets. The RSNN implemented on FPGA contains 8 input nodes, 4 LIF and 6 ALIF models in the hidden layer and 5 output nodes. In the input layer, the first 2 nodes are applied as inhibitory and others are excitatory. In the hidden layer, the first 3 LIF models are inhibitory and others are excitatory. If presynaptic neurons are inhibitory, the synaptic weights are clipped in (−1, 0). In a similar way, the synaptic weights are clipped in (0, 1) when presynaptic neurons are excitatory. A buffer is implemented on FPGA to delay outputs of neurons. When the counter reaches the number of inputs, the enable port of neurons is set to 0 and the update enable is set to 1. Then, the synaptic weights are updated.

The learning system is implemented based on 24-bit fixed-point data, considering the accuracy and consumption of computing resources. The energy and resource consumption of operations based on different data are shown in Table 2 (Horowitz, 2014). It shows that operations of fixed-point data cost fewer energy and area than floating point data. A multiplier for 16-bit floating data requires 2 DSP blocks or 51 look-up tables (LUTs) and 95 flip-flops (FFs) with a maximum working frequency of 219MHz. But a multiplier for 16-bit fixed-point data only requires 1 DSP block with a maximum working frequency of 300MHz. The 24-bit fixed-point used in this study is described as:


(25) $${\left(-1\right)}^{s\,i\,g\,n}\times \left(i\,n\,t\,e\,g\,e\,r+f\,r\,a\,c\,t\,i\,o\,n/2^{16}\right)$$

**TABLE 2:** Energy and area consumption of data operations.

Operations	Energy (pJ)	Area (μm^2^)	Operations	Energy (pJ)	Area (μm^2^)
8-bit fixed Add	0.03	36	32-bit fixed Add	0.1	137
8-bit fixed Mult	0.2	282	32-bit fixed Mult	3.1	3,495
16-bit floating Add	0.4	1,360	32-bit floating Add	0.9	4,184
16-bit floating Mult	1.1	1,640	32-bit floating Mult	3.7	7,700

where the 0-15th bits are the fraction part of data, the 16–22nd bits are the integer part of data and the 23rd bit is the sign of data.

### ALIF model implementation

**FIGURE 3:**
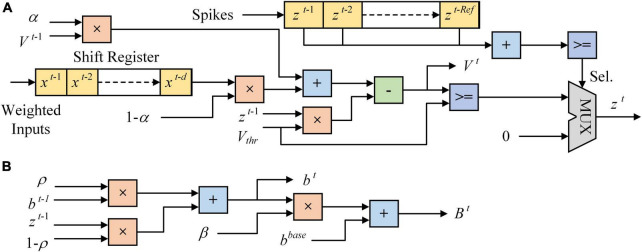
Architecture of neuron models implemented on FPGA. **(A)** LIF model. **(B)** Dynamic threshold in ALIF model.

Figure 3 shows the architecture of LIF and ALIF models implemented on FPGA. In Figure 3A, a 24-bit shift register and a 1-bit shift register are implemented in a LIF model as synaptic delay. When the clock increases 1, data is shifted in registers and a new spike is output. The MUX module is used as a selector, which has three input ports and an output port. When the Sel. is 0, data in the first input port is chosen to be output. When the Sel. is 1, which means that the model is in the refractory period, 0 is chosen to be output. Figure 3B shows the architecture of dynamic threshold voltage. In the ALIF model, *V*_*thr*_ in Figure 3A is replaced by *B^t^*.

**FIGURE 4:**
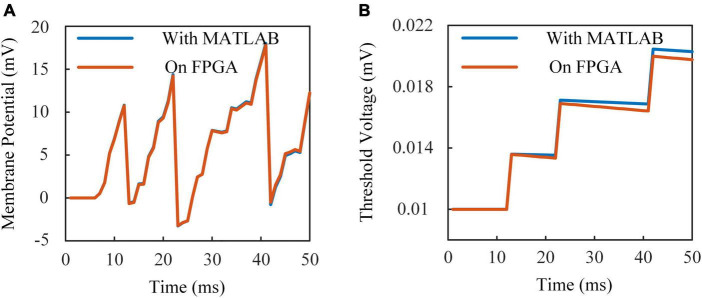
Responses of ALIF model implemented on FPGA and simulated with MATLAB with the same inputs. **(A)** Membrane potential. **(B)** Threshold voltage.

Operations of fixed-point data reduce energy and resource consumptions with a little bit loss in accuracy. Figure 4A shows the membrane potential of ALIF models simulated on a computer and implemented on FPGA. Figure 4B shows the dynamic threshold voltage of ALIF models with devices. Error evaluation is applied to ALIF model with four criteria, including mean absolute error (MAE), minimum root-mean-square error (RMSE), correlation coefficient (CORR) and R-square (*R*^2^) described as:


(26) $$M\,A\,E=\frac{1}{N}\,\sum_{i=1}^{N}\left|X_{s\,o\,f}\,\left(i\right)-X_{h\,a\,r}\,\left(i\right)\right|$$

(27) $$R\,M\,S\,E=\sqrt{\frac{1}{N}\,\sum_{i=1}^{N}{\left(X_{s\,o\,f}\,\left(i\right)-X_{h\,a\,r}\,\left(i\right)\right)}^{2}}$$

(28) $$C\,O\,R\,R=\frac{c\,o\,v\,\left(X_{s\,o\,f},X_{h\,a\,r}\right)}{\sigma \,\left(X_{s\,o\,f}\right)\,\sigma \,\left(X_{h\,a\,r}\right)}$$

(29) $$R^{2}=1-\frac{\sum_{I=1}^{N}{\left(X_{s\,o\,f}\,\left(i\right)-X_{h\,a\,r}\,\left(i\right)\right)}^{2}}{\sum_{I=1}^{N}{\left({\bar{X}}_{s\,o\,f}-X_{s\,o\,f}\,\left(i\right)\right)}^{2}}$$

where *X*_*sof*_ and *X*_*har*_ are results of simulation with MATLAB and implementation on FPGA, *N* is the number of data for error evaluation, and ${\bar{X}}_{s\,o\,f}$ is the mean value of *X*_*sof*_, CORR is the ratio of covariance to two data sets computed as:


(30) $$c\,o\,v\,\left(X_{s\,o\,f},X_{h\,a\,r}\right)=\sum_{i=1}^{N}\left(X_{s\,o\,f}\,\left(i\right)-{\bar{X}}_{s\,o\,f}\right)\,\left(X_{h\,a\,r}\,\left(i\right)-{\bar{X}}_{h\,a\,r}\right)$$

(31) $$\sigma \,\left(x\right)=\sqrt{\sum_{i=1}^{N}{\left(x\,\left(i\right)-\bar{x}\right)}^{2}}$$

where ${\bar{X}}_{h\,a\,r}$ is the mean value of *X*_*har*_. Results of error evaluation are shown in Table 3.

**TABLE 3:** Error evaluation results.

	MAE	RMSE	*R* ^2^	CORR
Membrane potential	9.1667 × 10^–5^	1.1723 × 10^–4^	0.9995	0.9999
Dynamic threshold	2.3894 × 10^–4^	3.0328 × 10^–4^	0.9930	0.9999

### Presynaptic spike-driven plasticity

The synaptic module based on eligibility trace is implemented on FPGA to train the RSNN. The learning rule is composed of three factors: presynaptic activities, postsynaptic activities and errors. Errors and the postsynaptic activities are instantaneous information. Presynaptic activities are stored in the buffer, which requires a lot of registers. The presynaptic spike-driven architecture is used to reduce the registers in buffers. Different with buffers used for inputs, a counter with a FF and a MUX selector is implemented as the buffer of synaptic module. In this way, hundreds of synaptic modules require fewer resources.

**FIGURE 5:**
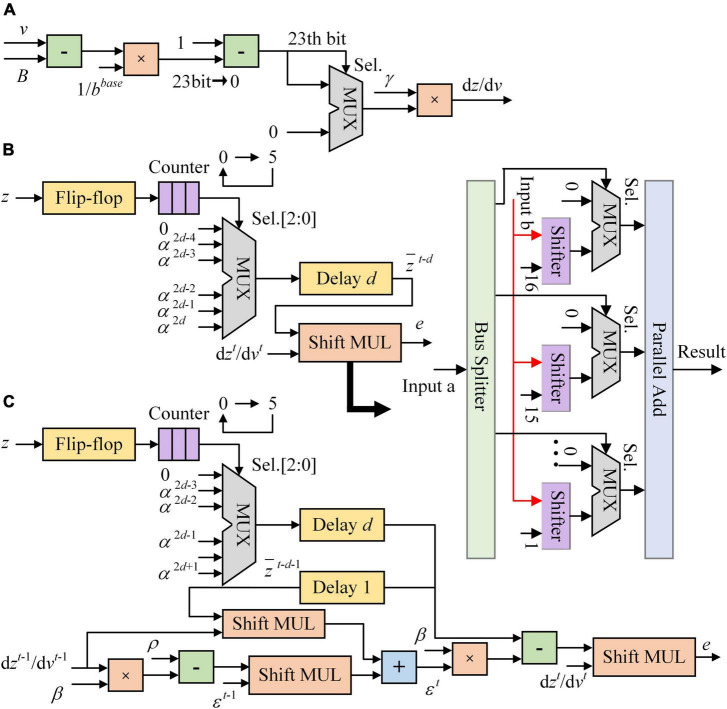
The architecture of synaptic module implemented on FPGA. **(A)** Pseudo-derivative of Heaviside function. **(B)** Eligibility trace of LIF model. **(C)** Eligibility trace of ALIF model.

Figure 5 shows the architecture of synaptic module implemented on FPGA. The pseudo-derivative of Heaviside function is limited to 0–0.3 as shown in Figure 5A. The eligibility traces of LIF and ALIF models use Shift MUL modules as multipliers and driven by presynaptic spikes as shown in Figures 5B, C. Because the refractory period is equal to the time window of eligibility traces, there is at most one spike in 5 timesteps. The FF is activated when a presynaptic spike arrives. If the number in counter reaches 5 (the length of time window), the counter is reset to 0. At each timestep, the counter outputs the number to the MUX selector. Then, the MUX selector outputs the constant data in input ports according to the three-bit selector signal in the Sel. port. In Figure 5B, shift and addition operations are used to replace multiplication between a constant and a variable. Besides, the synaptic module consists of the Shift MUL module, which is used as a multiplier between two variables. The “Input a” of Shift MUL is expected to be 0-1 which matches the scale of inputs. The 16–23rd bits of “Input a” are dropped and the 0-15th bits are split to 16 MUX selectors as control signals in Sel. ports. “Input b” is input to 16 shifters and shifted right from 1 to 16 bits. The first input port of MUX selectors is set to 0. When the Sel. port is 0, the MUX selector outputs 0. The second input port of MUX selectors is corresponding to the split data of “Input a.” If the 0th bit of “Input a” is input to the MUX selector, the “Input b” is input to this selector after shifted right 16 bits. If the 15th bit of “Input a” is input to the MUX selector, the “Input b” is input to this selector after shifted right 1 bit. When the Sel. port is 1, the MUX selector outputs the number in the second input port. If the synaptic weight in the module is positive, it is clipped in (0, 1). If the synaptic weight in the module is negative, it is clipped in (−1, 0). Besides, presynaptic spike-driven plasticity module, a regular synaptic module is designed based on shift registers as buffers to compare with the module based on presynaptic spike-driven architecture. The resource utilizations of these two modules are shown in Table 4. In the regular synaptic module, five 24-bit registers are used to store the attenuated spikes. This buffer requires times of resources of the buffer that is based on five single-bit LUTs and a selector. The presynaptic spike-driven plasticity module requires less resources on FPGA than the regular module. It reduces 38.9% LUTs, 49.5% registers, and 34.8% dynamic power consumptions. For an on-chip learning system implemented on FPGA, there are hundreds or thousands of synapses in RSNN. It greatly contributes to the high-efficient performance of learning system.

**TABLE 4:** Resource utilization of synaptic plasticity.

	LUTs	Registers	Dynamic power (mW)	Static power (mW)
Spike-driven	1976	229	54.96	902.55
Regular	3232	453	84.26	903

### Classifier implementation

When the last pixel is input to the RSNN, output nodes are activated by SoftMax function, which is used as a classifier. The SoftMax function reduces the complexity of gradients of the output weights. It normalizes the outputs of RSNN and then maps them to the possibility of predicted labels. The output node with the maximum probability becomes the prediction of RSNN. The SoftMax function is described as:


(32) $$S_{i}=\frac{e^{i}}{\sum_{j}e^{j}}$$

**FIGURE 6:**
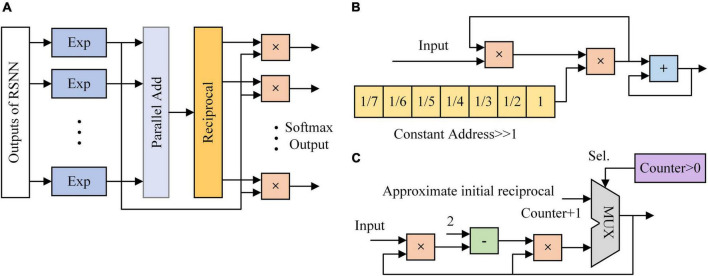
The architecture of classifier implemented on FPGA. **(A)** SoftMax function. **(B)** Exponent module. **(C)** Reciprocal module based on Newton-Raphson.

**FIGURE 7:**
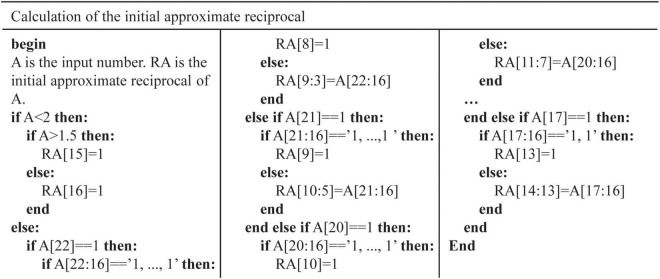
Pseudocode of initial approximate reciprocal value in the reciprocal module.

**FIGURE 8:**
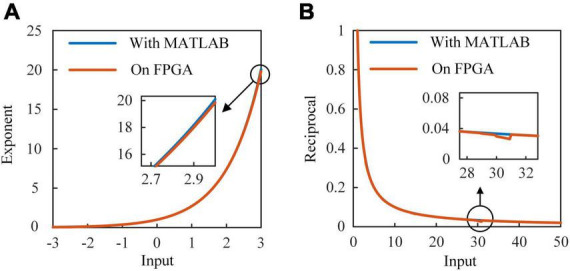
Results of exponent and reciprocal modules simulated in MATLAB and implemented on FPGA. **(A)** The exponent module on FPGA and EXP function in MATLAB. **(B)** The reciprocal module on FPGA and 1/x function in MATLAB.

Figure 6A shows the architecture of SoftMax function. It is mainly composed of the exponent module and the reciprocal module. Ten exponent (EXP) modules are implemented to calculate the exponents of outputs of RSNN. A parallel adder is used to sum outputs of EXP modules and transmit the result to the reciprocal module. Exponent results are multiplied with the reciprocal and become the probabilities of predicted labels. In Figure 6B, values 1/7, 1/6, …, 1 are stored in 7 24-bit registers. Once the adder and multipliers in the EXP module finish operations, the constant address of registers is shifted right 1. When the 7th value is input to the multiplier, the EXP module outputs the exponent result. Figure 6C shows the architecture of reciprocal module. The reciprocal module is designed based on Newton-Raphson (NR) method. The approximate reciprocal value is obtained by three cycles of calculation. The key problem of the implementation of Newton-Raphson method is how to get an accurate initial approximate reciprocal value. In this study, an architecture based on shift operation is proposed to find the initial approximate reciprocal value. The pseudocode of this method is shown in Figure 7. When a data is input to the reciprocal module, the highest bit with value 1 of the input data determines the shift operations. When data below this bit in the integer part are “1, …, 1,” the initial approximate reciprocal value is the same as the input data. If the data is between 1.5 and 2, the initial approximate reciprocal value is set to 1/2. If the data is smaller than 1.5, the initial approximate reciprocal value is set to 1. Since exponents are positive, the sign bit is set to 0. The same error evaluation is applied to the exponent module and reciprocal module. Figure 8A shows the exponent operation simulated in MATLAB and implemented on FPGA. Figure 8B shows the reciprocal module evaluation in the same way. The evaluation results are shown in Table 5.

**TABLE 5:** Error evaluation results.

	MAE	RMSE	*R* ^2^	CORR
Exponent	0.0155	0.0412	0.9999	1.0000
Reciprocal	2.9869 × 10^–4^	9.7972 × 10^–4^	0.9999	1.0000

## Results

### Results of experiments

**FIGURE 9:**
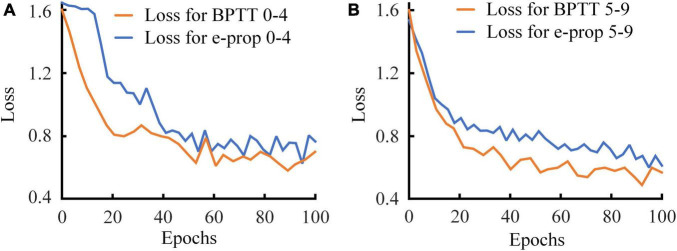
Loss value of RSNN based on BPTT and eligibility trace. **(A)** Half of the dataset consists of number 0–4 images. **(B)** Another half of the dataset consists of number 5–9 images.

Before implementing the RSNN on FPGA, we first test it on the computer based on the restricted e-prop algorithm and BPTT algorithm to confirm it converge to a similar loss value. We limit the RSNN to 8-10-5 nodes in view of the resources on FPGA. Accordingly, the MNIST dataset is divided into two parts. One includes images of number 0–4, and the other one is composed of images of number 5–9. The RSNN is trained on Xeon(R) Silver 4114 CPU for 100 epochs. We show the loss values of RSNNs at each epoch in Figure 9. In Figure 9A, the loss of network trained by BPTT algorithm based on 0-4 images decreases rapidly in the first 20 epochs. After 20 epochs, it enters a steady period. The loss of RSNN based on the e-prop algorithm decreases slower than the loss of BPTT algorithm. Trained after 40 epochs, the loss gradually stabilizes at a level slightly higher than the BPTT algorithm. Although it converges more slowly, it reaches a stable result with such a small size. In Figure 9B, the loss values of two RSNNs exhibit the same trend as in Figure 9A. The results of loss values in the training process show that the e-prop algorithm have similar convergence to the BPTT algorithm in such small-scale RSNNs.

**FIGURE 10:**
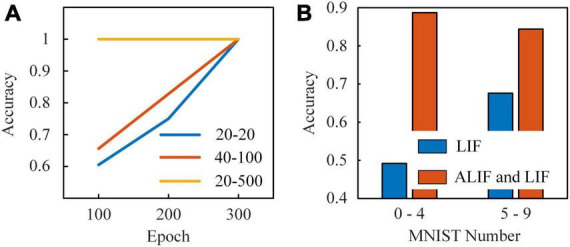
**(A)** Results of the learning system presented by five classes of spike patterns. The first number in the legend is τv. The second number in the legend is τa. **(B)** Results of the learning system with two kinds of neuron models and with only LIF model based on MNIST dataset.

After simulations on the computer, we implement this RSNN on Stratix V GX FPGA using Quartus Prime software. There are 8 input nodes, 10 neuron modules in the hidden layer and 5 output nodes in the RSNN. The input layer consists of 2 inhibitory and 6 excitatory LIF nodes, and the hidden layer includes 3 inhibitory LIF nodes, 1 excitatory LIF node and 6 excitatory ALIF nodes. Inhibitory models are coupled with inhibitory synaptic weights, which are limited to (−1, 0). Excitatory models are coupled with excitatory synaptic weights, which are limited to (0, 1). Synaptic modules are placed in each connection between neuron modules. We start tests with a spatio-temporal spike patterns classification task (Mohemmed et al., 2012). Spike trains with five spike patterns are presented sequentially to the RSNN. Each pattern is given by 8 random spike trains with a certain frequency distribution, which continues 900 timesteps. The RSNN is expected to map these input patterns to specific targets. We set three groups of *τ_*v*_* and *τ_*a*_* of ALIF models to explore how the dynamics in threshold voltage contributes to the learning ability. With *τ_*v*_* = 20 and *τ_*a*_* = 20, the membrane potential and dynamic threshold are in the same time scale. The threshold voltage decreases rapidly with the membrane potential after a spike generation in the ALIF model. Considering that neurons in the RSNN are activated sparsely, the threshold voltage decreases to the base value before the next activation, which means that ALIF models present no improvement in short-term memory. In Figure 10A, the RSNN with this group of *τ_*v*_* and *τ_*a*_* reaches an accuracy of 1 after trained 300 epochs. With *τ_*v*_* = 40 and *τ_*a*_* = 100, the membrane potential and dynamic threshold are in a similar time scale. The RSNN learns faster than the former network, but still requires at least 300 epochs to reach an accuracy of 1. With *τ_*v*_* = 20 and *τ_*a*_* = 500, the threshold voltage is at a much larger time-scale than the membrane potential. The slowly changing threshold enriches the inherent dynamics of ALIF models. As a result, the RSNN is stabilized at an accuracy of 1 trained after 100 epochs. The dynamic threshold voltage in a large time-scale makes up for inferior short-term memory capabilities in RSNNs.

Finally, we test the performance of RSNNs based on MNIST dataset. Since the RSNN implemented on FPGA only consists of 23 nodes and 180 synapses, the dataset is divided into two parts. One set includes images of number 0-4 and another one includes images of number 5–9. The pixels in each image are presented sequentially to input nodes, which have uniform increasing thresholds from 0.125 to 1. When the gray value of pixel is higher than the threshold, the input node generates a spike. Benefit from the reconfigurable neuron and synaptic modules in the learning system, the module types can be easily switched between LIF and ALIF neuron modules. Thus, we compare the classification accuracy of two RSNNs in Figure 10B. One RSNN only includes LIF models and another RSNN consists of LIF and ALIF models. The classification accuracy of the RSNN with LIF and ALIF models for each number in the 0–4 MNIST dataset is 97.9, 96.7, 82.6, 72.3, and 91.1%, respectively, which is 88, 81, 84.6, 79.1, and 88.7% in the 5–9 MNIST dataset. The total accuracy of the tests on these two datasets is 88.7 and 84.4%. In contrast, the accuracy of the RSNN with only LIF models for each number in the 0–4 MNIST dataset is 43.1, 54.3, 66, 26.1, and 44.6%, which is 62.5, 79.7, 48.3, 76.9, and 73.1% in the 5–9 MNIST dataset. The total accuracy of the tests is 49.2 and 67.6%, which is much lower than the RSNN with LIF and ALIF models. This comparison further confirms that the ALIF models greatly contribute to computational power of the RSNNs.

### Hardware consumption evaluation

The experiment results show that the learning system solves tasks accurately. A hardware consumption evaluation is then performed on the system to test the hardware efficiency. We measure the hardware consumption in FPGA in terms of LUTs, registers and power. In order to illustrate the advantages of the presynaptic spike-driven architecture, we compare the compilation results of our implementation of the RSNN with previous works based on other architectures. In Table 6, we show the resource utilization and power cost of three networks implemented on FPGA. The first implementation by Vo (2017) is a SNN trained by BP algorithm. Vo (2017) uses the pre-backpropagation block and backpropagation block to calculate errors and update synaptic weights in SNNs. The second one is a SNN implemented based on a clock-driven architecture proposed by Pani et al. (2017). The clock drives all neural models and synaptic modules to be updated at every simulation step, regardless of the spiking activity. Note that all results are normalized to the same network scale and the resource utilization does not include the external memories in this table. The LUTs utilization of our learning system is reduced to half of the implementation by Vo (2017). A more significant difference is in the usage of registers. Implementations by Vo (2017) and Pani et al. (2017) consume almost the same number of registers/slices because of the similar architecture they apply to SNNs. Only 18,081 registers are used in our architecture, which is almost 1/7 of theirs. This result suggests that our architecture performs excellent in resource utilization, especially in registers. Besides the resource utilizations, we also show the power cost in Table 6, which is estimated by the PowerPlay Power Analyzer Tool in Quartus Prime software. Although the static power consumption is different between FPGA development boards, our learning system consumes about 3.3W power less than the system proposed by Pani et al. (2017), which indicates that our learning system works at a low power level.

**TABLE 6:** Resource and power utilization of implementations for networks.

	LUTs	Registers/Slices	Power (W)	Platform
Vo (2017)	253,727	134,467	–	Spartan-3
Pani et al. (2017)	160,667	139,443	8.5	Virtex-6
This study	121,855	18,081	5.183	Stratix V GX

## Discussion

In this study, we use the restricted e-prop algorithm to train RSNNs, which updates the synaptic weights by surrogate gradients. This surrogate gradient is based on eligibility traces. Different from the global gradients backpropagated from the top layer, the eligibility trace represents the events of neurons, which means it is only related to a local spike. Based on this algorithm, we apply a presynaptic spike-driven architecture to the RSNN and implement it on FPGA. When a spike from presynaptic neuron arrives at the buffer, it activates this module to search the value of eligibility trace in a LUT. A learning signal from output layer is also used to guide the behaviors of neurons, which provides the global gradient to gate the eligibility trace.

Besides the spike-driven architecture, the time-driven architecture is also used to implement the RSNNs on FPGA. We compare the presynaptic spike-driven architecture with implementations by Vo (2017) and Pani et al. (2017) to illustrate the mechanisms of these two architectures, and discuss the possible sources of high resource and power consumptions of their works. The earlier studies generally focus on improving the throughput of systems to optimize accelerators. Vo (2017) uses backpropagation (BP) algorithm to train a small SNN on FPGA. Although the implementation presents satisfactory accuracy in test, a large amount of on-chip memories are used to store variables over time. The same problem exists in the clock-driven architecture proposed by Pani et al. (2017). The clock drives all neural models and synaptic modules to be updated at every simulation step, regardless of the spiking activity. As a results, many invalid activities and variables occupy the memories and computing resources. Even the neuron is in the refractory period, its output is also stored and the synaptic module is updated. In contrast, the synaptic modules in this study are activated sparsely and the activities of neurons are limited to timestamps. This intermittent activation mode makes the RSNN work in a lower energy manner than previous works. The power consumption in Table 6 suggests the high power-efficiency of our learning system.

In recent years, many event-driven/spike-driven architectures are proposed for implementations of SNNs on FPGA. Compared with time-driven/clock-driven architecture, spikes occupy a smaller bit width in the data transmission and storage between modules (Moore et al., 2012; Pani et al., 2017). This is reflected in the utilization of registers in Table 6. Sankaran et al. (2022) use the single spike control BPTT algorithm to train the RSNN and implement this on FPGA based on the event-driven architecture. This architecture depends on the number of spikes instead of spike-time information and weight values stored on on-chip memories. But it relies on the request-acknowledge cycles between layers to allow the layer’s time execution. The request-acknowledge cycles access information in each layer frequently. High-throughput data transmission and power consumption are both challenges in this architecture. Park and Jung (2020) use the latest timestamp and the synaptic modification rate to trace the exponential decay STDP function (Sim et al., 2019). This architecture converts the complex relationship between activities in pre and postsynaptic neurons to the timestamps in the address domain. It greatly contributes to the reduction of buffer size. Inspired by this, we use timestamps to represent traces of spikes instead of entire eligibility traces. The spike is simultaneously used as an enable signal for synaptic modules, which prevents all modules in RSNNs from updating at every time step. Only those synaptic modules that receive spikes are driven to be updated, which reduces the inefficient works. We confirm that the combination of eligibility traces and presynaptic spike-driven architecture can reduce the buffer size of synaptic modules, which leads to the reduction of resource utilization of the entire learning system.

## Conclusion

In this study, we realize a high-efficient RSNN learning system on FPGA with excellent software-to-hardware reproduction. This architecture is based on the spikes generated by RSNNs, which is compatible with FPGA. Meanwhile, it provides flexible reconfigurability for modifying the network connectivity, model types and other parameters. We provide several modules that simplify computation, such as the Shift MUL module and SoftMax module. We perform two inference applications to test the RSNNs implemented on FPGA, which are the spike patterns classification and the MNIST handwriting digits classification. In the former test, we implement ALIF models with three groups of parameters and explore how dynamics in threshold voltage contributes to the learning ability. In the latter test, we implement two RSNNs with different neuron modules and further confirm the contributions of ALIF models to the computational power of RSNNs. The compilation results and power estimation of RSNNs on FPGA show that the requirements of LUTs, registers and dynamic power consumptions of synaptic modules are respectively reduced by 38.9, 49.5, and 34.8%. The presynaptic spike-driven architecture contributes to reduce the resource utilization of the entire on-chip learning system while accurately solving the tasks, as the buffer size for caching events is greatly reduced. This architecture for RSNNs provides an alternative way for realizing the large-scale neuromorphic learning systems, as the transmission and storage of data on chips greatly limit the scale of systems (Li et al., 2015; Que et al., 2022). The spike-driven architecture may offer a solution for these problems.

## Data availability statement

Publicly available datasets were analyzed in this study. This data can be found here: http://yann.lecun.com/exdb/mnist/.

## Author contributions

All authors contributed to the different phases of the research and to the writing of this manuscript.

## References

[B1] Ahmad A. M. Ismail S. Samaon D. F. (2004). “Recurrent neural network with back propagation through time for speech recognition,” in *Proceedings of the IEEE international symposium on communications and information technology*, Sapporo. 10.1109/ISCIT.2004.1412458

[B2] Aljuaid A. Anwar M. (2022). Survey of supervised learning for medical image processing. *SN Comput. Sci.* 3:292. 10.1007/s42979-022-01166-1 35602289 PMC9112642

[B3] Bellec G. Salaj D. Subramoney A. Legenstein R. Maass W. (2018). “Long short-term memory and learning-to-learn in networks of spiking neurons,” in *Proceedings of the neural information processing systems (NeurIPS)*, Montréal, Canada. 10.5555/3326943.3327017

[B4] Bellec G. Scherr F. Hajek E. Salaj D. Subramoney A. Legenstein R. (2019). “Eligibility traces provide a data-inspired alternative to back propagation through time,” in *Proceedings of the neural information processing systems (NeurIPS)*, Vancouver, Canada.

[B5] Bellec G. Scherr F. Subramoney A. Hajek E. Salaj D. Legenstein R. (2020). A solution to the learning dilemma for recurrent networks of spiking neurons. *Nat. Commun.* 11:3625. 10.1038/s41467-020-17236-y 32681001 PMC7367848

[B6] Benda J. Herz A. V. M. (2003). A universal model for spike-frequency adaptation. *Neural Comput.* 15 2523–2564. 10.1162/089976603322385063 14577853

[B7] Benjamin B. V. Gao P. McQuinn E. Choudhary S. Chandrasekaran A. R. Bussat J. M. (2014). Neurogrid: A mixed-analog-digital multichip system for large-scale neural simulations. *Proc. IEEE* 102 699–716. 10.1109/JPROC.2014.2313565

[B8] Chu M. Kim B. Park S. Hwang H. Jeon M. Lee B. H. (2015). Neuromorphic hardware system for visual pattern recognition with memristor array and CMOS neuron. *IEEE Trans. Ind. Electron.* 62 2410–2419. 10.1109/TIE.2014.2356439

[B9] Davies M. Srinivasa N. Lin T. H. Chinya G. Cao Y. Choday S. H. (2018). Loihi: A neuromorphic manycore processor with on-chip learning. *IEEE Micro* 38 82–99. 10.1109/MM.2018.112130359

[B10] Dundar A. Jin J. Martini B. Culurciello E. (2017). Embedded streaming deep neural networks accelerator with applications. *IEEE Trans. Neural Netw. Learn. Syst.* 28 1572–1583. 10.1109/TNNLS.2016.2545298 27071200

[B11] Fieres J. Schemmel J. Meier K. (2008). “Realizing biological spiking network models in a configurable wafer-scale hardware system,” in *Proceedings of the IEEE international joint conference on neural networks (IEEE world congress on computational intelligence)*, Hong Kong. 10.1109/IJCNN.2008.4633916

[B12] Hermans M. Dambre J. Bienstman P. (2015). Optoelectronic systems trained with backpropagation through time. *IEEE Trans. Neural Netw. Learn. Syst.* 26 1545–1550. 10.1109/TNNLS.2014.2344002 25137733

[B13] Horowitz M. (2014). “1.1 Computing’s energy problem (and what we can do about it),” in *Proceedings of the IEEE international solid-state circuits conference digest of technical papers (ISSCC)*, San Francisco, CA. 10.1109/ISSCC.2014.6757323

[B14] Kaiser J. Mostafa H. Neftci E. (2020). Synaptic plasticity dynamics for deep continuous local learning (DECOLLE). *Front. Neurosci.* 14:424. 10.3389/fnins.2020.00424 32477050 PMC7235446

[B15] Kalhor E. Noori A. Noori G. (2021). Cancer cells population control in a delayed-model of a leukemic patient using the combination of the eligibility traces algorithm and neural networks. *Int. J. Mach. Learn. Cybern.* 12 1973–1992. 10.1007/s13042-021-01287-8

[B16] Kornijcuk V. Jeong D. S. (2019). Recent progress in real-time adaptable digital neuromorphic hardware. *Adv. Intell. Syst.* 1:1900030. 10.1002/aisy.201900030

[B17] Kriegeskorte N. Mok R. M. (2017). Building machines that adapt and compute like brains. *Behav. Brain Sci.* 40:269. 10.1017/S0140525X17000188 29342705

[B18] Larsen R. S. Sjöström P. J. (2015). Synapse-type-specific plasticity in local circuits. *Curr. Opin. Neurobiol.* 35 127–135. 10.1016/j.conb.2015.08.001 26310110 PMC5280068

[B19] Lechner M. Hasani R. Amini A. Henzinger T. A. Rus D. Grosu R. (2020). Neural circuit policies enabling auditable autonomy. *Nat. Mach. Intell.* 2 642–652. 10.1038/s42256-020-00237-3

[B20] Li S. Wu C. Li H. Li B. Wang Y. Qiu Q. (2015). “FPGA acceleration of recurrent neural network based language model,” in *Proceedings of the annual international symposium on field-programmable custom computing machines*, Vancouver, BC. 10.1109/FCCM.2015.50

[B21] Liu B. Ye X. Zhou C. Liu Y. Zhang Q. Dong F. (2020). “The improved algorithm of deep Q-learning network based on eligibility trace,” in *Proceedings of the international conference on control, automation and robotics (ICCAR)*, Singapore. 10.1109/ICCAR49639.2020.9108040

[B22] Manneschi L. Vasilaki E. (2020). An alternative to backpropagation through time. *Nat. Mach. Intell.* 2 155–156. 10.1038/s42256-020-0162-9

[B23] Merolla P. A. Arthur J. V. Alvarez-Icaza R. Cassidy A. S. Sawada J. Akopyan F. (2014). A million spiking-neuron integrated circuit with a scalable communication network and interface. *Science* 345 668–673. 10.1126/science.1254642 25104385

[B24] Millner S. Grübl A. Meier K. Schemmel J. Schwartz M. O. (2010). A VLSI implementation of the adaptive exponential integrate-and-fire neuron model. *Adv. Neural Inf. Process. Syst.* 2 1642–1650. 10.5555/2997046.2997079

[B25] Mohemmed A. Schliebs S. Matsuda S. Kasabov N. (2012). SPAN: Spike pattern association neuron for learning spatio-temporal spike patterns. *Int. J. Neural Syst.* 22 1659–1685. 10.1142/S0129065712500128 22830962

[B26] Moore S. W. Fox P. J. Marsh S. J. Markettos A. T. Mujumdar A. (2012). “Bluehive – A field-programable custom computing machine for extreme-scale real-time neural network simulation,” in *Proceedings of the international symposium on field-programmable custom computing machines*, Toronto, ON. 10.1109/FCCM.2012.32

[B27] Painkras E. Plana L. A. Garside J. Temple S. Galluppi F. Patterson C. (2013). SpiNNaker: A 1-w 18-core system-on-chip for massively-parallel neural network simulation. *IEEE J. Solid State Circuits* 48 1943–1953. 10.1109/JSSC.2013.2259038

[B28] Pani D. Meloni P. Tuveri G. Palumbo F. Massobrio P. Raffo L. (2017). An FPGA platform for real-time simulation of spiking neuronal networks. *Front. Neurosci.* 11:90. 10.3389/fnins.2017.00090 28293163 PMC5328944

[B29] Park J. Jung S. D. (2020). Presynaptic spike-driven spike timing-dependent plasticity with address event representation for large-scale neuromorphic systems. *IEEE Trans. Circuits Syst. I* 67 1936–1947. 10.1109/TCSI.2020.2966884

[B30] Que Z. Nakahara H. Nurvitadhi E. Boutros A. Fan H. Zeng C. (2022). Recurrent neural networks with column-wise matrix–vector multiplication on FPGAs. *IEEE Trans. Very Large Scale Integr. (VLSI) Syst.* 30 227–237. 10.1109/TVLSI.2021.3135353

[B31] Salaj D. Subramoney A. Kraisnikovic C. Bellec G. Legenstein R. Maass W. (2021). Spike frequency adaptation supports network computations on emporally dispersed information. *ELife* 10:e65459. 10.7554/eLife.65459 34310281 PMC8313230

[B32] Sankaran A. Detterer P. Kannan K. Alachiotis N. Corradi F. (2022). “An event-driven recurrent spiking neural network architecture for efficient inference on FPGA,” in *Proceedings of the international conference on neuromorphic systems*, Knoxville, TN, United States. 10.1145/3546790.3546802

[B33] Schwenker F. Trentin E. (2014). Partially supervised learning for pattern recognition. *Pattern Recognit. Lett.* 37 1–3. 10.1016/j.patrec.2013.10.014

[B34] Shama F. Haghiri S. Imani M. A. (2020). FPGA realization of Hodgkin-Huxley neuronal model. *IEEE Trans. Neural Syst. Rehabil. Eng.* 28 1059–1068. 10.1109/TNSRE.2020.2980475 32175866

[B35] Sim J. Joo S. Jung S. O. (2019). “Comparative analysis of digital STDP learning circuits designed using counter and shift register,” in *Proceedings of the international technical conference on circuits/systems, computers and communications (ITC-CSCC)*, JeJu. 10.1109/ITC-CSCC.2019.8793424

[B36] Sutton R. S. Barto A. G. (2014). *Reinforcement learning an introduction second edition.* Cambridge, MA: MIT Press.

[B37] Tang H. Glass J. (2018). “On training recurrent networks with truncated backpropagation through time in speech recognition,” in *Proceedings of the IEEE spoken language technology workshop*, Cambridge, MA. 10.1109/SLT.2018.8639517

[B38] Vo H. M. (2017). “Implementing the on-chip backpropagation learning algorithm on FPGA architecture,” in *Proceedings of the international conference on system science & engineering*, Ho Chi Minh City. 10.1007/s11265-005-4961-3

[B39] Wang X. Liu Y. Sanchez-Vives M. V. McCormick D. A. (2003). Adaptation and temporal decorrelation by single neurons in the primary visual cortex. *J. Neurophysiol.* 89 3279–3293. 10.1152/jn.00242.2003 12649312

[B40] Werbos P. J. (1990). Backpropagation through time: What it does and how to do it. *Proc. IEEE.* 78 1550–1560. 10.1109/5.58337

[B41] Zhang W. Li P. (2019). “Spike-train level backpropagation for training deep recurrent spiking neural networks,” in *Proceedings of the neural information processing systems (NeurIPS)*, Vancouver, Canada. 10.5555/3454287.3454988

[B42] Zhou Y. Ren Y. Xu E. Liu S. Zhou L. (2022). Supervised semantic segmentation based on deep learning: a survey. *Multimedia Tools Appl.* 81 29283–29304. 10.1007/s11042-022-12842-y

